# Recherches Sur L’Introduction Accidentelle De L’Air Dans Les Veines, &c.

**Published:** 1840-08-08

**Authors:** 


### MEDICAL EXAMINER.

DEVOTED TO MEDICINE, SURGERY, AND THE COLLATERAL SCIENCES.

No. 32.] PHILADELPHIA, SATURDAY, AUGUST 8, 1840. [Vol. III.

### BIBLIOGRAPHICAL NOTICE.

*Recherches sur l'Introduction Accidentelle de l'Air dans les Veines, &c.* Par J. Z. Amussat. Paris, 1839.

*Researches on the Accidental Introduction of Air into the Veins; and especially on the Question, "Can air introduced spontaneously by a wounded vein during a surgical operation cause death suddenly?"* By J. Z. Amussat. Paris, 1839. 8vo. pp. 250.

The subject of M. Amussat's monograph has for some years past been exciting considerable and increasing interest. In the early part of the present century, the alarming effects produced by the accidental introduction of air into the jugular vein of a horse, during the operation of bleeding, at the veterinary school of Alfort, directed attention to the question comprehended in the title of M. Amussat's work. Subsequently, the occurrence of well marked and fatal accidents, from analogous causes in the human subject, arrested the special notice of surgeons and physiologists, among them M. Magendie, from whom it elicited the publication of a lecture on the subject, as well as M. Amussat, who made some researches, submitted to the Academy in 1835, in a Memoir on Traumatic Hæmorrhages. M. Amussat has since followed up the subject in the treatise lately published, in which he collects all the facts known on the subject, and discusses them in a calm, impartial, and elaborate style. We are indebted to the July number of the Edinburgh Medical and Surgical Journal, for a very complete notice of M. Amussat's work, from which we condense a tolerably full summary of the leading facts and important conclusions.

The cases recorded by M. Amussat are thirty-nine in number, which may be classified in the following manner:—

"The whole thirty-nine facts naturally arrange themselves in five distinct classes or categories. The first category consists of ten facts, which may be called incontestible, in so far as their correctness receives confirmation from the evidence of dissection. The second category contains six facts analogous to the preceding, but without the evidence of dissection. The third contains fourteen instances of air admitted into the veins, twelve in man and two in animals, and in which recovery took place. The fourth division contains seven doubtful facts. And the fifth contains two instances of suicide produced by wound in the neck, and in which there was strong reason to believe death had been the result of the ingress of air into the veins.

"Viewed in the relation of the diseases and operations, these 39 cases resolve themselves into the following divisions.

"1. Nine instances of tumour of the neck; 2. seven instances of operation on the shoulder or the arm-pit; viz. two cases of tumour of the right shoulder; three cases of extirpation of the arm; and two tumours of the axillary region; 3. six cases of tumour of the breast; 4. two cases of ligature of the subclavian artery; 5. four cases of tumour of the face; 6. five instances of blood-letting from the jugular vein, two in man, and three in the horse; 7. four cases of wounds of the jugular vein, (suicidal;) and 8. two cases without positive indication of the nature of the operation.

"Considering the cases as to the veins by which the air has been introduced, the following are the results.

"1. By wound of the external jugular vein, the phenomenon took place four times; 2. by the external jugular vein, twelve times; four times by blood-letting in the neck; 3. by the veins of the neck canalized, that is rendered open continuous canals, four times; (see pages 208 and 210;) 4. by the subclavian vein, once; 5. by the axillary vein, three times; 6. by the subscapular vein, twice; 7. by a vein opening into the subclavian, once; 8. by the pectoral veins, three times; 9. by the facial vein once; and, 10. six or eight times, the veins opened have not been specified.

"As to the duration and the stage of the operations, death took place eight times at the close of long and laborious operations, which weakened the patients by hemorrhagy and nervous exhaustion; and especially at the termination of the operation, at the moment when the tumour is removed.

"Twice, however, death took place in consequence of the accident occurring at the commencement of the operation. Death, it must be further remarked, is more prompt when the patients are most enfeebled, as was seen in the cases which occurred to M. Delpech, M. Castara, and M. Roux.

"As to the duration of the accident, we find that, among sixteen deaths, the authors of the cases say, eight in nine times, that death took place suddenly, without specifying the actual duration.

"In one case (Ulrich,) death took place one minute after the accident; in one case (Beauchene,) forty-five minutes after; in one case (Clemot,) some hours after; in one case (Mirault,) three hours and a half after; in one case (Roux,) seven days after the accident.

"In many cases of recovery it is almost always at the commencement of the operation that the accident has occurred. In the case recorded by M. Amussat himself, however, the accident took place at the end of a long and laborious operation. In this case, however, he observes little air entered, because the vessel was small, and the opening was promptly closed.

"There is, however, a very obvious reason for the accident occurring more commonly at the close of an operation than at its commencement. It is then chiefly that the deep-seated vessels are divided; it is then, above all, that those vessels are most usually divided, which are most directly connected with the veins of the chest.

"M. Amussat remarks as to sex and age, that the cases give seventeen men from 21 to 60 years; seventeen females from 18 to 68; two cases in which the age and sex are not specified; and three horses. It must be observed on this head, that it is not very philosophical to trace any connection in this inquiry between the ages of the patients, or even the sex, and the accident. It is quite clear, that it is the primary disorder that ought, in such circumstances to be kept in view. Tumours of the neck or shoulder requiring operation may occur at any age. Most of the cases of tumours of the neck were examples of encephaloma; those of the shoulder were either of that nature, or *osteo-sarcoma;* and the tumours in the breast and axilla appear to have been scirrhus or carcinoma.

"In those circumstances it is almost superfluous for M. Amussat to say, that it is interesting to observe, that the phenomenon has not been observed in children, and to add, that the reason is perhaps because at that age there its less frequent occasion to perform operations in the dangerous region.

"II. Though the collection of facts already adduced may be regarded as competent to establish the truth of the general inference, that air may, in certain circumstances, be introduced into the veins of the animal body, and that, when introduced, it speedily gives rise to most pernicious and commonly mortal effects, yet there are various other points which require either confirmation or some elucidation. Of this kind are the questions, which are the circumstances favourable or unfavourable to the introduction of air into the venous system? why is air inspired or drawn into the veins of one part of the body and not into those of another? and are there any known means by which this accident may be prevented, or if it take place, its pernicious effects may be counteracted? To throw some light on, if not to determine these questions, is the great object of the experimental inquiry undertaken by M. Amussat.

"M. Amussat divides his inquiry into three chapters; the first relating to the spontaneous introduction of air into the veins; the second relating to the forced or artificial introduction of air into the venous system; and the third devoted to the examination of the means of preventing or arresting the accident, or counteracting its effects.

"A. M. Amussat observes, in the first place, in reference to the spontaneous introduction of air into the veins, that his researches show that air cannot be introduced spontaneously into all veins. These researches show that this phenomenon can take place in the normal state only in those veins in which there is a reflux of blood, or what is designated by the name of the venous pulse (*pulsus venosus.*) On this subject Haller may be consulted, lib. vi. sect. iv. It is sufficient to say, that these veins are all those which are situate at the anterior part of the neck, at the upper part of the chest, and in the arm pit. The space in which these accidents are likely to take place, and to which he applies the name of *dangerous*, may be circumscribed by two semi-elliptical lines, going from one arm-pit to the other, the one above the collar-bones, the other below them. But if the veins be supposed to be canalized or converted into cylindrical tubes like the arteries, the phenomenon might take place over a much larger extent.

"The experiments performed by M. Amussat are of three orders. In the first are placed all those experiments which relate to the spontaneous introduction of air into the veins of animals in the normal state. In the second are placed all those experiments which demonstrate the influence of the abstraction of a certain quantity of blood before the production of the spontaneous phenomenon. And in the third division, he proposes to prove that, by converting the veins into uncollapsing canals, the spontaneous phenomenon may be produced at a much greater distance from the heart, than is observed in the normal state.

"These experiments were performed on rabbits, dogs, sheep, and horses, by exposing one or other jugular vein, or the axillary vein, and thus allowing the air to enter without any additional contrivance. In the second series of experiments, quantities of blood, varying from a few ounces to some pounds, according to the size of the animal, were previously withdrawn. In the third series of experiments, M. Amussat introduced a caoutchouc tube into the interior of the veins, and in this manner rendered these tissues inflexible or incapable of collapsing. Our limits do not allow us to detail these experiments; but we give the results obtained in each order of cases.

"Of the first series of experiments, the following summary gives the general results.

"It may be regarded as established, that the spontaneous introduction of air by a vein wounded near the apex of the chest, in animals of different sizes, produces almost always death in a manner more or less sudden.

"Among twenty-six animals subjected to this experiment 24 died, 2 resisted. The rabbits died in the course of from one minute and a half, to 2, 3, or 5 minutes. The dogs died in the course of one minute, 3, 6, 10, 16, 24, and 27 minutes. Two survived. The sheep died in the course of from 19 to 56 minutes. The horses died in the course of 14, 15, 17, 26, 28, 35 minutes, 1 hour and 44 minutes, 1 hour and 50 minutes, and 2 hours and 30 minutes. One animal alone resisted, because, having fallen with the neck supported against a tree, there was formed a clot which plugged up the opening in the vein. But when the clot was removed, he died in 26 minutes afterwards.

"As a general rule, the duration of each experiment is included between the moment of the introduction of the air and death. As soon as the eyelids begin to flutter, when a finger is placed on the animal's eye, it is understood that life has then ceased.

"In almost all these experiments the right cavities of the heart alone contained frothy blood. The lungs were sound. The veins of the brain contained seldom some bells of air.

"B. Of the second series of experiments, the following summary gives the results.

"It appeared clearly that depletion, by withdrawing a certain quantity of blood from the vessels, has considerable influence on the promptitude with which the effects of the spontaneous introduction of air takes place. Among 16 animals subjected to this kind of experiment, 14 died; one alone, a dog, resisted its effects. In another dog some bells of air were allowed to enter; and he was destroyed in order to ascertain whether the air had been admitted. The dogs died in the course of 1, 4, 6, and 25 minutes; the sheep after 7 and 16 minutes; the horses after 3 minutes and a half, 9, 12, 13, 16, and 23 minutes.

"In some cases, as in the second experiment, on the horse, the phenomena approach with as great rapidity as in the case of the forced and sudden insufflation. The symptoms are the same; death is as speedy; and, on inspection, air is also found in the left cavities. This difference can be explained only by the facility with which the air passes through the vessels, suddenly emptied. Death takes place doubtless more speedily, because the three principal organs are injured by the presence of air in the vessels.

"To conclude, it may be said that the spontaneous introduction of air by a wounded vein, determines death so much more quickly, as the animal has lost a greater quantity of blood, or as he has been exhausted by pain. It is doubtful whether debility from any other cause can produce the same result. It might appear at first sight, that debility from fatigue, old age, or disease, ought to be equivalent in influence to the abstraction of a certain quantity of blood. But the experiments upon horses, most of which were diseased, prove that it is not those which are most feeble in appearance that resist the least. A horse that could scarcely stand from absolute debility, survived the commencement of the experiment 16 minutes.

"C. The third series of experiments shows that if a tube be introduced into a vein beyond the point of reflux of the blood, or, in other words, beyond the dangerous region, and carried into the dangerous region through the vein, air may be in this manner also introduced into the superior cava and heart, and the usual effects will follow. It appears, in short, from these experiments, that, if a wounded vein be dilated and kept open like an artery by an organic disease, the phenomenon of the spontaneous introduction of air is almost as much to be dreaded, as if the vein were wounded within the limits of the venous pulse.

"In the second chapter, M. Amussat details the experiments on the forced or artificial introduction of air into the veins. Air may be introduced into veins in this mode, either suddenly or slowly. In the first case it almost uniformly produces speedy and irrecoverable death, as is well known by the experiments of Nysten. The results of the experiments performed by M. Amussat may be stated in the following manner.

"Among 18 animals subjected to this sort of experiment, 15 died. Rabbits died in the course of from 50 seconds to 1 minute; dogs in the course of 2 minutes; sheep in from 1 to 2 minutes; and horses in the course of 3, 5, 6, 6½, 13, and 16 minutes. Three dogs resisted the experiment, which was performed by injecting air with a syringe.

"The symptoms observed are nearly the same in all. Whatever be the animal, death is instantaneous. He falls as if struck by lightnight, or stunned by the blow of a mace. Scarcely is there time to observe what passes between the instant of the forced introduction of air and death.

"Sometimes, however, the animals resist longer, 16 minutes, for instance, as seen in the last experiments upon the horses. But in these cases the insufflation had been made gently, and by a person whose lungs were of small capacity.

Another source of difference in results may also be the circumstance, that in some cases it was deemed unnecessary to tie the veins on the tube employed for insufflation; and when this is neglected, it may happen that the air returns between the *parietes* of the vein and the tube.

"In all cases the heart is distended with frothy blood; and the venous and arterial vessels are more or less filled with air.

"Death takes place evidently from the interruption of the circulation. The air distends the right chambers of the heart, and prevents the venous blood from reaching them.

"When the air is introduced forcibly but slowly, the phenomena are quite the same; but the result is longer in taking place, and death consequently is slower in approaching. This depends on the gradual introduction of air, because then the harmony of the functions of respiration and circulation is not suddenly destroyed. In general, a larger quantity of air is required to produce the effect, when it is impelled gently into the venous system; and in some instances, it does not in this mode produce the fatal termination.

"On comparing these last results with those of the first series, that is, those of the spontaneous introduction of air into the veins, it is seen that there is a great analogy between the phenomena which ensue when air is impelled gently, and when it is introduced spontaneously. The symptoms are the same; and the animals die nearly in the same space of time.

"There is nevertheless this remarkable difference, that after death produced by the spontaneous introduction of air, in general the right chambers only of the heart are distended with air, whereas, after its forcible introduction, air is found in the left chambers as well in the arteries as the veins.

"The difference in the rate of rapidity with which death takes place, may be explained by saying, that, in the forcible introduction of air, the functions of the heart, of the lungs, and of the brain are disturbed, while in the case of slow or spontaneous introduction, the disturbance is confined to the two first organs.

"D. These experiments, and the results obtained by them, lead naturally to the third object of inquiry, namely, to ascertain whether we possess any means of preventing or arresting this formidable accident, or, after it takes place, of counteracting its effects. Though the experiments undertaken with the first two objects are calculated to throw some light on this question, M. Amussat has devoted to its express elucidation ten experiments; and the results of these may be stated in the following manner.

"It appears, that it is not possible to prevent the occurrence of this accident by previously compressing the chest and belly, as has been imagined. The only compression which is efficacious is that which is applied directly over the vein, or the principal venous trunk; and the best means of remedying as much as possible the accident, is to close quickly the aperture by which the air has obtained admission. By observing this precaution, the continuance of the phenomenon is prevented, and the animal may survive, if too much air has not been introduced. The same result sometimes takes place when a clot is formed in the aperture.

"When much air has obtained admission, and the animal resists its effects, if frothy blood escapes at each expiration, it is a favourable symptom; for if the opening be closed, the animal, which might have lived, dies speedily.

"M. Amussat concludes, therefore, that the experiments detailed in the present essay lead naturally to the use of the means which he has specified, and which consist in stopping at first the aperture, or causing the patient to make quickly motions of inspiration, and in favouring this time of respiration by compressing the chest and belly, in order to expel the air introduced.

"There is, however, in this statement a singular inconsistency, which may lead to an important and serious practical error. M. Amussat says, that the means of arresting the accident consist in first stopping the aperture, and causing the patient to perform motions of expiration. Now, to any one who knows anything either of this accident, or of the influence of respiration on its occurrence, it must appear absurd and impracticable to speak of *closing* the aperture, and causing the patient to make efforts of *expiration*. If efforts of expiration be made while the aperture is closed, not the slightest benefit will result; and if it be wished to prevent the accident from going further, the aperture should be open, while the patient makes efforts of expiration. It is during *inspiration* that the aperture ought to be closed, because it is during inspiration that the air is liable to be drawn in by a vein situate within the influence of the function of respiration. There is no risk of air being drawn in during *expiration*, but some risk lest it may not be expelled if already admitted. The rule, therefore, ought to be, instead of first closing the aperture, and directing the patient to make efforts of expiration, to close the aperture the moment the hissing sound is heard, and keep it closed during *inspiration*; but if air have been admitted, to open it during *expiration*, in order to favour the escape of air, which always comes away in the form of frothy blood. It must be observed, nevertheless, that, unless this can be done with perfect accuracy, the best and safest course is to keep the aperture constantly shut, as the slightest mistake in opening the aperture during *inspiration* instead of *expiration*, may, by giving admission to a large quantity of air, prove instantly fatal. When it is kept closed, there is no chance of further admission to air; and if that already admitted be not very great, it is absorbed, and the circulation is re-established. This result, there is good reason to believe, took place in those instances of recovery in which air had obtained admission into the venous system.

"M. Amussat further thinks, that the best means of counteracting the effects of the accidental admission of air into the venous system consists in pumping out the air introduced into the heart; and that it is requisite to introduce promptly a tube, in order to make aspiration, that is, exhaustion of the air, as it passes rapidly into the ventricle, and even into the vascular system. This practice may be successful; but we cannot help looking upon it as exceedingly dangerous, and likely to be detrimental. The chance of extracting air in this manner is small and precarious; and, unless the utmost care be taken, air is as likely to be admitted as extracted. M. Amussat himself allows that the animal sometimes dies, before there is time to operate, when all the preparations are previously made.

"The general result of the whole of the experimental researches detailed in this part of his work, M. Amussat comprehends in the following fifteen aphorisms, which we annex as presenting a condensed view of the conclusions of the whole matter.

"1. The spontaneous introduction of air by an opening made in a vein in the neighbourhood of the lower part of the neck, and upper part of the chest, where the reflux of the venous blood is observed, is a constant phenomenon.

"2. Air introduced in this manner produces almost always a peculiar sound, which cannot be mistaken, when once heard, and which it is difficult to confound with any other noise, because it has peculiar characters.

"3. The intensity of the phenomenon is in proportion to the aperture in the vein, its size, the vicinity to the heart, and especially the force of inspiration.

"4. The danger of the introduction of air into the veins is greater in proportion to the quantity of blood lost by the animal.

"5. Death is so much the more speedy, in proportion as the animal has suffered long.

"6. On opening the chest of animals dead suddenly by the spontaneous introduction of air into the veins, the right chambers of the heart are found constantly distended, inflated with air, more or less mixed with blood, while the left chambers are almost always empty, collapsed, and contain little or no air.

"7. The cause of death appears especially to be attributable to the interruption of the pulmonary circulation.

"8. The vertical position often favours the introduction of air, because the animal is agitated, and makes great inspirations.

"9. The phenomenon may take place beyond its legitimate limits, fixed by the reflux of the blood, by canalizing the vein by means of a tube.

"10. The sudden and forcible introduction of air into the veins produces almost invariably instant death, in animals very different in species and size.

"11. The previous compression of the chest and of the abdomen does not prevent the occurrence of the spontaneous introduction of air.

"12. The aspiration of air by a wounded vein is produced altogether by the thoracic walls, and in no degree either by the heart or lungs.

"13. Air escapes during expiration in all the positions in which an animal is placed; but in the standing attitude, the blood remains in the wound, is there coagulated, and plugs up the aperture.

"14. Compression of the chest and belly after expiration favours the issue of the air, and by repeating this manœuvre several times the heart is aided in ridding itself of the air which distends its right chambers.

"15. Lastly, aspiration by a glass tube and syringe shows that more blood than air is withdrawn; but the blood may be repelled, retaining the air, and the operation may be resumed, so as to aspire almost all the air contained in the right chambers of the heart.

"Upon these aphorisms, we have nothing to observe, further than what has been already said on the stoppage of the wound during inspiration, and the probable dangerous effects of attempting aspiration.

"III. In the third part of his essay, M. Amussat undertakes to give an explanation of the phenomenon of the spontaneous introduction of air into the veins.

He first proceeds to answer any objection that might be urged against the fact of the presence of air in the right chambers of the heart, by showing that air, in such circumstances, could not proceed from decomposition, or be introduced by incautious opening of the cavities. It is always possible to distinguish between air generated under such circumstances and that introduced accidentally, by the presence of other marks of decomposition, and by the circumstance of air being found in other cavities and parts of the body. In this accident, on the other hand, the right chambers only are distended with air mixed with blood; the blood also is frothy, if the body be examined immediately or soon after the accident; and the left chambers are almost always empty. It is impossible, therefore, to confound the appearances with cadaveric effects.

"Another proof which shows that it is impossible to confound the circumstances and effects of the accidental introduction of air into the veins during life with cadaveric appearances, even with the presence of a large quantity of air in the heart, is obtained from performing on a dead body the following experiment. If, after opening one of the jugular or the subclavian veins very near the chest, alternate pressure be made on the walls of this cavity, in order to imitate respiration, then air obtains admission, because the chest performs the function of a sucking pump. If the chest and pericardium be opened carefully, the right chambers are found distended and inflated with air, and the left chambers are in their usual state. Upon opening the right chambers air escapes in a body, and they collapse. On opening the left chambers, nothing of the kind is observed. In this case the right chambers are distended with air, but the air is not mixed with blood. The blood is not frothy; an essential character which establishes a difference between air introduced during life and air introduced after death.

"The knowledge of these facts is of importance in a medico-legal point of view.

"From all the facts described by the experiments now mentioned, it results almost inevitably, that the introduction of air into a wounded vein near the upper part of the chest is a physical and almost purely mechanical phenomenon. It is a mechanical effect of the action of bellows, produced by the external pressure of the air on the right chambers of the heart. The alternate motion of the chest determines the entrance of the air; and it does so by acting on veins which are not, by their position, allowed to collapse. The adherence of the venous parietes to their cellular sheaths, where they form canals, and where these sheaths adhere to bones, as in the case of the subclavian veins, and the reflux of the blood by the motion of respiration, are the two causes which determine the admission of air into the wounded veins near the upper part of the chest.

"He very properly remarks, that the action of the auricle, which in propelling the blood produces an oscillation in that column which regurgitates in the *venæ cavæ*, performs no part in the introduction of air into the veins, in which the venous pulse is observed.

"It is known that this name is applied to the reflux of blood which is observed in the jugular and subclavian vein during expiration, and which depends on the temporary impediment given to the progress of the blood in the *venæ cavæ* at the moment at which expiration takes place, and the lungs sink, and the blood of the pulmonary artery is consequently prevented from continuing its progress. This refluent motion or mere stationary condition of blood during inspiration is very distinct in the jugular veins of emaciated persons, when the breathing is difficult or embarrassed, in disease of the heart, and in persons in the last agonies.

"The phenomena of the venous pulse or the reflux of blood may be easily observed in animals. It is distinctly seen in the jugular and subclavian veins, at some distance from the chest, in the swelling of these vessels. In great motions of expiration, the reflux ascends to some height, when the circulating currents are unimpeded; but when the blood is prevented from moving, the reflux ascends only one or two inches above the first rib.

"M. Amussat further thinks that this reflux is particularly produced by the compression of the right auricle, and of the *venæ cavæ*, by the lung during the motion of expiration. This cause we must regard as very doubtful in its operation. The lung may compress the auricle and the *venæ cavæ;* but would this compression produce the effect? The cause which appears to us much more efficient is that already assigned, namely, the temporary but sudden stop which the blood of the pulmonary artery encounters during expiration and the collapse of the lung, and which has the effect of interrupting the progress of the blood through the ventricle, auricle, and *venæ cavæ* at once.

"Before quitting this part of the subject, it is requisite to notice that a different hypothesis, both as to the cause of the introduction of the air, and as to the mode in which its presence produces death, has been recently propounded by Sir Charles Bell.

"The admission of air, Sir Charles ascribes, in the first instance, to the elevation of the muscles of the neck, by which the atmospheric pressure is taken off, and the air is drawn into the open vein. This, he conceives, is proved by the fact, that if in the dead subject an incision be made in the lower part of the neck, the external jugular vein is found flat or collapsed; but if an incision be made on raising the shoulder and collar-bone of that side, the vein opens; and if any fluid be poured into the hollow space of the neck, and the motions of respiration be imitated by alternately raising and depressing the shoulder and collar-bone, the fluid gradually descends into the vein.

"It is probable that the elevation of the shoulder and clavicle does take off pressure from the vein; but it seems also impossible to doubt that it has the effect of opening the vein and thereby canalizing it.

"From observing the very rapid manner in which death takes place in consequence of this accident, Sir Charles infers that it is the effect of the air entering the vertebral arteries, thus depriving the *medulla oblongata* of blood, and immediately thereby extinguishing in its source, all motion, voice, and expression. A doctrine very similar had been previously taught, first, by Bichat in a general manner, regarding the brain being the organ first affected, and afterwards more particularly as to the *medulla oblongata* being disordered in its circulation, by Dr. Handyside.

"IV. The fourth part of the essay is devoted to the practical deductions. These have been in a great degree already anticipated; and the necessity of drawing these observations to a close prevents us from dwelling on the subject.

"It is only in the case of operations on the neck, the upper part of the chest, the arm-pit, and the shoulder, that this accident is really to be dreaded. And it is more particularly immediately above and immediately below the collar-bone, in a circumscribed space, where the reflux of the blood or the venous pulse is observed, that it most usually happens. Particular circumstances, however, as the adhesion of a tumour to a vein, rigidity of the surrounding parts, and similar causes, may extend the sphere of danger, so that incisions beyond the boundary specified may be followed by the introduction of air into the veins.

"Under all circumstances, the best rule that can be laid down as preventive consists in compressing the principal venous trunk, between the heart and the part on which the operation is to be performed.

"The application of this rule to particular cases constitutes the great difficulty in practice. In the case of venesection from the jugular vein, it is necessary to avoid opening that vessel low down, within reach of the reflux of the blood, and it is further requisite that the wound in the skin and that in the vein be quite parallel.

"M. Amussat expresses an apprehension that the dread of this accident may deter timid and cautious surgeons from attempting important operations in the neck and shoulder, or in the arm-pit. He sees reason also to blame the surgeons of small localities for excessive reserve, lest professional jealousy should take an opportunity of proclaiming any unsuccessful results. It cannot be denied that M. Amussat has good grounds both for the apprehension and the censure; and it too often happens that a rash and ignorant confidence will urge one man to undertake operations, which others more modest and timid, but better informed and better qualified, would have declined. The remedy for both is knowledge, and especially that knowledge recommended by M. Amussat, which is obtained by performing the operation, or, at least, imitating its performance on the lower animals several times, before it be attempted on the human subject. As to the extreme reserve deprecated by the author, and the cause to which he ascribes it, it is unfortunately a dark page in the history of surgeons, that too often any mishap is blazoned with the most benevolent zeal and assiduity,—sometimes with a reasonable amount of commiseration for the man who has been so unfortunate or so ill-advised, while every favourable case is allowed to pass in silence and obscurity. For this there is no remedy, except to recommend every one who censures, to consider what he would or could have done in similar circumstances; and if he be conscientious, he will be sparing in the language of censure or criticism.

"In a sort of supplementary note, M. Amussat adverts to sudden deaths after delivery, which may have proceeded from the admission of air into the veins. It is a known fact, that females have died immediately or soon after child-birth, in circumstances which rendered it impossible to ascribe the event to hæmorrhage, the most usual cause of death at that time. M. Chomel states in one of his lectures, that, in the course of six years, he knew that among four hundred and fifty individuals dead at La Charité, seven died in an unexpected manner, while the most minute examination after death did not disclose any thing which could explain the sudden and fatal termination. In these cases, he conjectures that the cause might have been the admission of bells of air from the womb into the blood-vessels. Similar facts appear to have occurred in the practice of Baudelocque."

